# Cooperative Interfacial and Electrolyte Engineering in Au@Cu_2_O Core@Shell Nanocatalysts for Efficient CO_2_ Reduction to C_2+_ Products at Circumneutral pH

**DOI:** 10.1002/smll.76442

**Published:** 2026-10-24

**Authors:** Eunice Estrella De Guzman, Jen‐An Shih, Wan‐Ru Lee, Yung‐Jung Hsu, Chia‐Yu Lin

**Affiliations:** ^1^ Department of Chemical Engineering National Cheng Kung University Tainan City Taiwan; ^2^ Department of Materials Science and Engineering National Yang Ming Chiao Tung University Hsinchu Taiwan; ^3^ Center for Emergent Functional Matter Science National Yang Ming Chiao Tung University Hsinchu Taiwan; ^4^ Institute of Integrated Research Institute of Science Tokyo Kanagawa Japan; ^5^ Center for Resilience and Intelligence on Sustainable Energy Research (RiSER) National Cheng Kung University Tainan Taiwan; ^6^ Program on Key Materials & Program on Smart and Sustainable Manufacturing Academy of Innovative Semiconductor and Sustainable Manufacturing National Cheng Kung University Tainan Taiwan; ^7^ Department of Chemical Engineering Faculty of Engineering Chulalongkorn University Bangkok Thailand

**Keywords:** carbon mitigation, C─C coupling, CO_2_ utilization, electrocatalysis, halide promotion, surface restructuring

## Abstract

Developing advanced catalytic systems for efficient electrochemical CO_2_ reduction (*e*‐CO_2_RR) at industrially relevant current densities under environmentally benign conditions remains challenging. Herein, we report Au@Cu_2_O (*n*) core@shell nanocatalysts with outstanding *e*‐CO_2_RR performance at ‐200 mA cm^−2^ in near‐neutral media, where the Cu_2_O shell thickness is tuned by adjusting Au seed solution volume (*n* mL). In 1.0 M KHCO_3_, the thin‐shelled Au@Cu_2_O (*n* = 5) nanocatalyst achieves an exceptional Faradaic efficiency (FE_C2+_: 50.6 ± 3.3%) and a cathodic energy efficiency (EE_C2+_: 29.1 ± 1.3%) for C_2+_ products, outperforming its thicker counterparts (e.g., Au@Cu_2_O (*n* = 2); FE_C2+_: 19.6 ± 2.7%, EE_C2+_: 8.9 ± 0.9%) and individual control components. This enhanced performance stems from a strong core@shell electron transfer effect that modulates copper oxidation state (OS_Cu_) and preserves the core@shell architecture. Intriguingly, iodide addition triggers divergent, shell‐thickness‐dependent effects. Iodide modulates both structural reconstruction and OS_Cu_ of the Au@Cu_2_O (*n* = 2) nanocatalyst, boosting C_2+_ product selectivity (S_C2+_) fourfold. Conversely, iodide disrupts the pre‐existing, highly favorable core@shell electronic interactions of the Au@Cu_2_O (*n* = 5) nanocatalyst, driving OS_Cu_ away from the favorable regime and thereby suppressing S_C2+_. These findings underscore the importance of cooperatively engineering interfacial electronic properties and electrolyte microenvironments for efficient *e*‐CO_2_RR at circumneutral pH.

## Graphical abstract

The e‐CO_2_RR performance of Au@Cu_2_O core–shell electrocatalysts is regulated by a cooperative interplay between shell thickness and electrolyte environments. In blank electrolyte, thin‐shelled variants leverage interfacial electronic synergy to optimize the copper oxidation state (OS_Cu_) and maximize C_2+_ selectivity. Intriguingly, iodide introduction optimizes OS_Cu_ and boosts C_2+_ selectivity for thick‐shell variants while driving them off‐optimum for thin‐shelled counterpart.

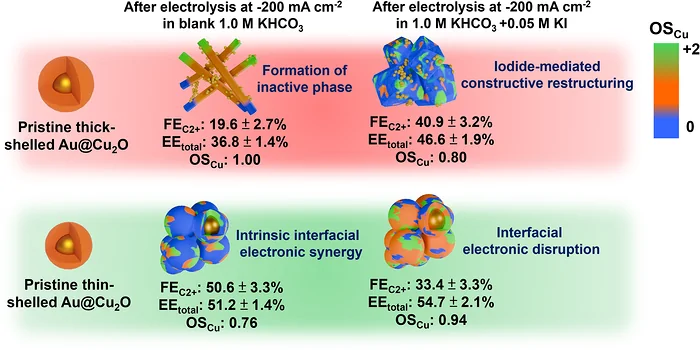

## 1 Introduction

Electrochemical CO_2_ reduction (*e*‐CO_2_RR) powered by renewable electricity offers a sustainable paradigm for transforming greenhouse gas CO_2_ into value‐added commodity chemicals and high‐energy fuels, simultaneously addressing carbon mitigation, green chemical synthesis, and renewable energy storage [1, 2]. Nevertheless, the thermodynamic stability and chemical inertness of the CO_2_ molecule, coupled with the intricate multi‐step proton‐coupled electron transfer pathways inherent to *e*‐CO_2_RR, typically result in sluggish reaction kinetics and severe parasitic side reactions [3]. Consequently, achieving high product selectivity, especially for high energy‐density and economic value multicarbon (C_2+_) products under environmentally benign, circumneutral pH conditions, remains a formidable challenge [4]. To render *e*‐CO_2_RR economically viable, the rational development of robust, efficient electrocatalysts capable of selectively steering pathways toward targeted products with minimal energy inputs is imperative.

To date, copper has been recognized as the uniquely effective element for driving the production of C_2+_ products with satisfactory Faradaic efficiency (FE_C2+_) [5, 6, 7, 8, 9, 10, 11]. The enhanced selectivity heavily relies on the synergistic catalysis of mixed‐valence Cu^+^/Cu^0^ pairs, wherein metallic Cu^0^ species activate CO_2_ to facilitate subsequent electron transfer, while Cu^+^ species strengthen the adsorption of *CO intermediate, accelerating C─C coupling and the subsequent formation of C_2+_ products [12, 13, 14, 15, 16, 17, 18, 19, 20, 21]. Nonetheless, during the course of *e*‐CO_2_RR, the oxidation state of surface copper species (OS_Cu_) is highly dynamic, leading to severe morphological restructuring of the catalyst and degradation of active sites [15, 21, 22]. This continuous in situ transformation severely deteriorates product selectivity and stability, highlighting the critical importance of controlling and stabilizing OS_Cu_ during operation *e*‐CO_2_RR to unlock sustained catalytic performance.

Several strategies have been explored to regulate the OS_Cu_ during the *e*‐CO_2_RR process, including the introduction of halide ions during the electrolysis [21, 23, 24, 25, 26] and the integration of Cu species with complementary metals assembled into specialized bimetallic geometries [27, 28, 29, 30, 31, 32]. For example, Sari et al. recently reported that the presence of an optimal concentration of iodide ions effectively regulates OS_Cu_ by facilitating the in situ transformation of CuI microcrystals into Cu^0^/Cu_2_O nanoaggregates. This architecture, featuring metallic Cu^0^ domains embedded within a crystalline Cu_2_O matrix, provides dual sites for both CO_2_ activation and C−C coupling, thereby significantly enhancing operational stability during prolonged electrolysis at −100 mA cm^−2^ [21]. However, most investigations concerning halide effects on the *e*‐CO_2_RR performance of the Cu‐based electrocatalytic systems have been restricted to low current regimes (< −100 mA cm^−2^), frequently at the expense of high operating overpotentials and the requirement of strongly alkaline microenvironments [33, 34, 35, 36].

On the other hand, Cu_2_O‐based bimetallic heterostructures emerge as a compelling architectural strategy to modulate the OS_Cu_ from a structural perspective. This spatially separated architecture enables intense interfacial electronic coupling between the distinct foreign metal and Cu_2_O domains, thereby modulating the OS_Cu_. For example, Wang et al. designed tandem catalysts featuring Au nanoparticles anchored on Cu_2_O surfaces with selective exposure of (100) and (111) facets to investigate *CO spillover dynamics. They discovered that a stronger interfacial interaction between the Au nanoparticles and Cu_2_O (100) reduced the energy barrier for *CO spillover, while the Cu_2_O (100) facet exhibits a reduced energy barrier for C─C coupling, consequently enhancing the selectivity toward C_2+_ products [37]. Although this tandem system demonstrated high selectivity for C_2+_ products in near‐neutral media (0.1 M KHCO_3_), it suffered from high operating overpotentials and a severely limited catalytic current density (e.g., ∼ −6.5 mA cm^−2^ at −1.2 V vs. RHE).

In the present contribution, we report a systematic study on Au@Cu_2_O core@shell nanocatalysts to elucidate how shell thickness and electrolyte additives cooperatively govern high‐rate *e*‐CO_2_RR at an industrially relevant current density of ‐200 mA cm^−2^ in near‐neutral media. The Cu_2_O shell thickness is tuned by adjusting the volume of Au seed solution (*n* mL) during the synthesis. Our findings demonstrate that the Au@Cu_2_O (*n* = 5) nanocatalyst, featuring a thin shell of ∼ 11.4 nm, exhibits superior intrinsic performance in a blank 1.0 M KHCO_3_ electrolyte, delivering a high Faradaic efficiency (FE_C2+_: 50.6 ± 3.2%) for C_2+_ products and a promising total cathodic energy efficiency (EE_total_: 51.2 ± 1.4%). Characterization indicates that alongside local hydrodynamic and microenvironmental factors (e.g., local alkalinization and mass transport at ‐200 mA cm^−2^), strong interfacial electronic interactions play a major role in modulating the average surface oxidation state (OS_Cu_: ∼ +0.76) and preserving the structural integrity of the core@shell architecture against phase deactivation into inactive malachite (Cu_2_CO_3_(OH)_2_). Conversely, the thicker shell (19.0 ± 2.0 nm) of the Au@Cu_2_O (*n* = 2) variant leads to an unfavorable OS_Cu_ of ∼1.00, causing the Cu_2_O shell to reconstruct into a microrod matrix containing the inactive malachite phase. Intriguingly, the introduction of iodide ions completely inverts these catalytic pathways as a function of shell dimensions, uncovering a dynamic dual modulation beyond simple static thickness correlations. For the thicker‐shelled Au@Cu_2_O (*n* = 2) nanocatalyst, iodide adsorption acts synergistically to drive a favorable structural reconstruction into active Au@CuO_x_ nanocubes while lowering OS_Cu_ toward a more favorable regime (+0.80), thereby dramatically boosting S_C2+_ and suppressing overpotentials. In sharp contrast, for the thin‐shelled Au@Cu_2_O (*n* = 5) catalyst, strong iodide adsorption disrupts the pre‐existing, highly favorable core@shell electronic interactions, driving the OS_Cu_ away from the favorable regime to ∼+0.94 and thereby suppressing the production of C_2+_ products. Nevertheless, iodide universally functions as an efficient interfacial kinetic promoter that substantially mitigates charge‐transfer resistance, lowering operating potentials across all configurations and yielding an exceptional peak EE_total_ (54.7 ± 2.1%) for the GDE|Au@Cu_2_O (*n* = 5) system. Coupled with its outstanding operational durability, the GDE|Au@Cu_2_O (*n* = 5) electrode stands out as a state‐of‐the‐art AuCu bimetallic electrocatalyst for high‐rate *e*‐CO_2_RR in near‐neutral media.

## 2 Results and Discussion

### 2.1 Materials Synthesis and Characterizations

The Au@Cu_2_O (*n*) nanocatalysts were synthesized according to our previously reported method, where the Cu_2_O shell thickness was modulated by adjusting the volume (*n* mL, *n* = 1, 2, 3, 4, 5) of Au core seed solution (0.25 mM) added to a solution containing a fixed concentration of Cu^2+^ ions (See Experimental Section for the details) [38]. XRD analyses (Figure S1a) confirm the presence of crystalline Cu_2_O and Au in all synthesized nanocatalysts. Notably, as the *n* value increases, a systematic simultaneous attenuation of the Cu_2_O diffraction peaks and a corresponding enhancement of the Au diffraction peaks are observed. Furthermore, SEM analyses (Figures S1b–f and S2) reveal that the overall sizes of the Au@Cu_2_O (*n* = 1), Au@Cu_2_O (*n* = 2), Au@Cu_2_O (*n* = 3), Au@Cu_2_O (*n* = 4), and Au@Cu_2_O (*n* = 5) nanocatalysts are 95.8 ± 3.0 nm, 74.0 ± 3.1 nm, 64.2 ± 1.9 nm, 53.7 ± 3.2 nm, and 44.0 ± 2.3 nm, respectively. Taken together, these XRD and SEM findings indicate that higher Au core seed content leads to a thinner Cu_2_O shell and smaller overall particle dimensions [27]. This structural evolution was further corroborated by TEM and HRTEM analyses (Figure 1a,b). TEM imaging (Figure 1a,b) shows the Cu_2_O shell thickness of Au@Cu_2_O (*n* = 2) is 19.0 ± 2.0 nm, which is thicker than the shell observed for Au@Cu_2_O (*n* = 5) (11.4 ± 2.5 nm). High‐resolution analysis reveals distinct lattice fringes for both samples. The *d*‐spacings of 0.21 and 0.24 nm correspond to (200) and (111) planes of Cu_2_O (JCPDS no. 05–0657), respectively. Additionally, a *d*‐spacing of 0.24 nm was observed in the core region, consistent with the (111) plane of metallic Au (JCPDS no. 04–0784), further validating the successful formation of the Au@Cu_2_O heterostructure. XPS analyses (Figure 1c,d) reveal that the Au@Cu_2_O (*n* = 5) nanocatalyst exhibited features characteristic of both Cu_2_O (a peak at 932.3 eV in the Cu 2p_3/2_ region) and metallic Au (peaks at binding energies of 83.8 eV and 87.5 eV with a separation of 3.7 eV in the Au 4f region), confirming the presence of both phases. In contrast, the Au@Cu_2_O (*n* = 2) nanocatalyst only displays a peak at 931.8 eV in the Cu 2p_3/2_ region, with no detectable Au^0^ signal. The absence of the Au signal for the Au@Cu_2_O (*n* = 2) nanocatalyst would be attributed to the greater Cu_2_O shell thickness of the Au@Cu_2_O (*n* = 2) nanocatalyst, which exceeds the characteristic XPS sampling depth of 5–10 nm [27, 39]. To confirm the core@shell geometry and probe the buried interface, XPS depth profiling with controlled Ar^+^ ion etching was conducted for the Au@Cu_2_O (*n* = 2) nanocatalyst (Figure S3). The depth‐resolved spectra reveal that the signal from the metallic Au core (Au 4f) becomes prominent after ≥ 5 s of Ar^+^ etching. Notably, concomitant with the appearance of the Au core signal, the binding energy of the peak in the Cu 2 p_3/2_ region shifts from 931.8 to 932.2 eV. This depth‐dependent spectral modulation provides direct experimental confirmation of the core@shell geometry and substantiates the presence of strong interfacial electronic interactions at the Au|Cu_2_O heterojunction. These findings further support the conclusion that the Au@Cu_2_O (*n* = 2) nanocatalyst possesses a thicker Cu_2_O shell compared to the Au@Cu_2_O (*n* = 5) sample. Furthermore, the binding energy of the Cu 2p_3/2_ peak for the Au@Cu_2_O (*n* = 5) nanocatalyst (932.2 eV) shifts toward a higher value compared to that of the Au@Cu_2_O (*n* = 2) sample (931.8 eV). Additionally, the binding energy of the Au^0^ peak in the Au 4f_7/2_ region (83.8 eV) is lower than values typically reported in the literature (∼84.2 eV, referenced to C1s peak at 284.8 eV) [40, 41]. AES spectra (Figure S4) exhibit three characteristic peaks at 568.0, 570.3, and 573.5 eV, assignable to Cu^0^, Cu^2+^/Cu^+^, and the transition states of the Cu LMM spectrum, respectively [11, 42]. Quantitative deconvolution of surface copper species reveals that the pristine GDE|Au@Cu_2_O (*n* = 2) and GDE|Au@Cu_2_O (*n* = 5) electrodes consisted of 26.9% Cu^0^, 46.4% Cu^+^, and 26.7% Cu^2+^, corresponding to an average oxidation state (OS_Cu_) of 0.79 and 0.89, respectively. These quantitative AES findings confirm that the thinner Au@Cu_2_O (*n* = 5) nanocatalyst has a higher average copper oxidation state, strongly substantiating the presence of pronounced core–shell interfacial electron transfer between the metallic Au core and the thin Cu_2_O shell.

**FIGURE 1:**
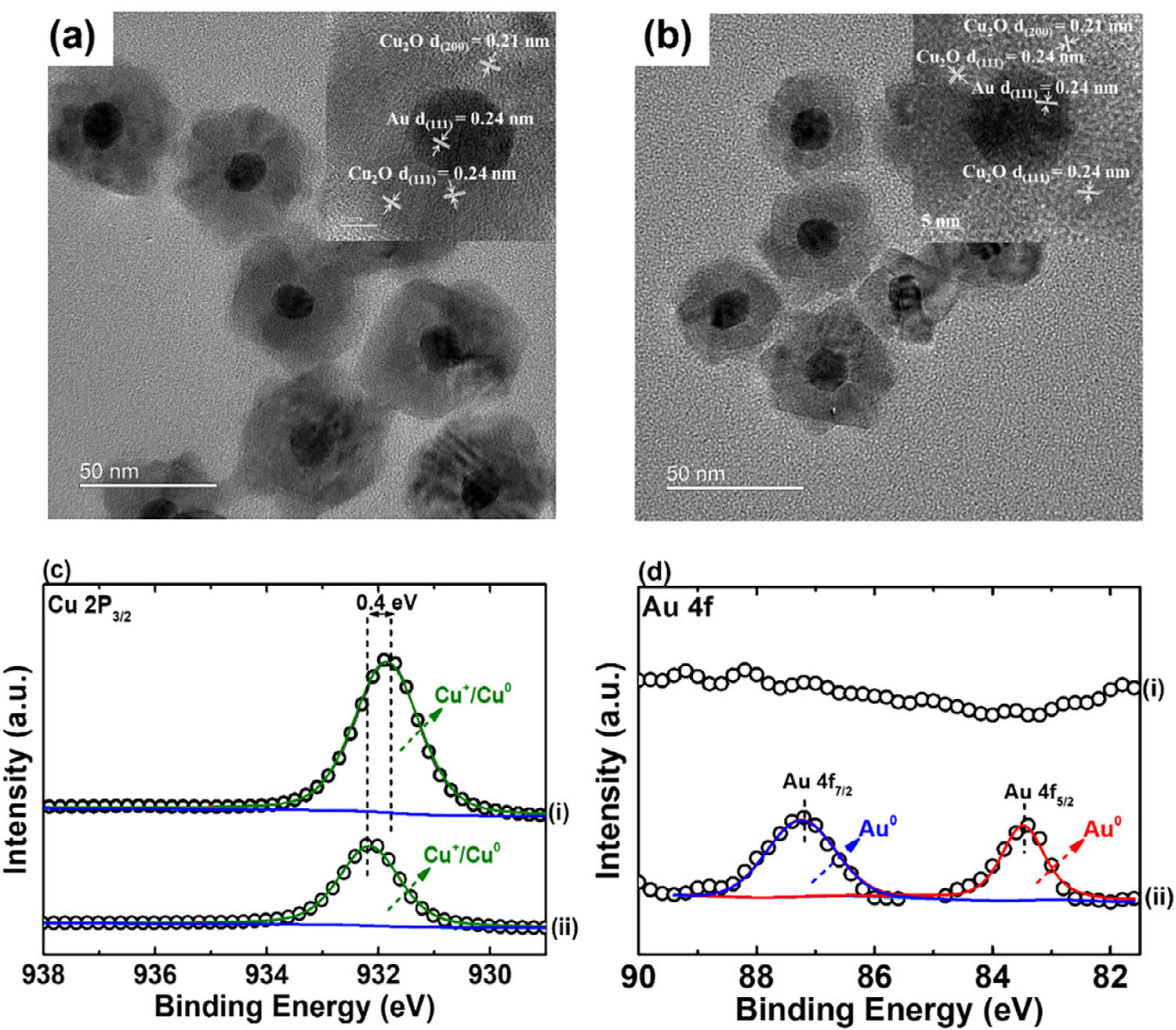
Structural characterization of Au@Cu_2_O (*n*) nanocatalysts. (a,b) TEM images of the (a) Au@Cu_2_O (*n* = 2) and (b) Au@Cu_2_O (*n* = 5) nanocatalysts; insets show the corresponding HR‐TEM images. High‐resolution XPS spectra of (c) Cu 2p_3/2_ and (d) Au 4f regions for (i) Au@Cu_2_O (*n* = 2) and (ii) Au@Cu_2_O (*n* = 5).

Figure 2a–c shows the electrochemical characterization of the *e*‐CO_2_RR performance for the GDE|Au@Cu_2_O (*n*) electrodes at −200 mA cm^−2^ in a blank 1.0 M KHCO_3_ solution. As shown in Figure 2a, the operating potentials of the GDE|Au@Cu_2_O (*n* = 1), GDE|Au@Cu_2_O (*n* = 2), GDE|Au@Cu_2_O (*n* = 3), GDE|Au@Cu_2_O (*n* = 4), and GDE|Au@Cu_2_O (*n* = 5) electrodes to sustain the applied current (i.e., ‐200 mA cm^−2^) are −1.39, −1.30, −1.18, −0.93, and −0.77 V vs. RHE, respectively. These findings suggest that overpotential decreases significantly as the Cu_2_O shell thickness is reduced. As revealed from the EIS analyses (Figure S5a and Table S1), the substantial reduction in overpotential with a thinner Cu_2_O shell stems directly from a significantly reduced charge‐transfer resistance (R_ct_). Specifically, the fitted R_ct_ values for the GDE|Au@Cu_2_O (*n* = 1), GDE|Au@Cu_2_O (*n* = 2), GDE|Au@Cu_2_O (*n* = 3), GDE|Au@Cu_2_O (*n* = 4), and GDE|Au@Cu_2_O (*n* = 5) electrodes are 6635 Ω, 4900 Ω, 4000 Ω, 1850 Ω, and 1200 Ω, respectively. Product analyses (Figure 2b,c) reveal that all prepared electrodes exhibited a total Faradaic efficiency (FE_total_) exceeding 80% for all generated *e*‐CO_2_RR products. The primary *e*‐CO_2_RR products were identified as formate, CO, ethanol, ethylene, and 1‐propanol, with the remaining ∼20% of the Faradaic efficiency being attributed to the competing HER. While FE_total_ remained high across all samples, the product distribution shifted notably with shell thickness. Specifically, the selectivity toward C_2+_ products (S_C2+_) increased as the Cu_2_O shell thinned. The S_C2+_ of the GDE|Au@Cu_2_O (*n* = 5) electrode reached 20.0%, which is about four‐fold higher than that of the GDE|Au@Cu_2_O (*n* = 2) electrode (4.8%).

**FIGURE 2:**
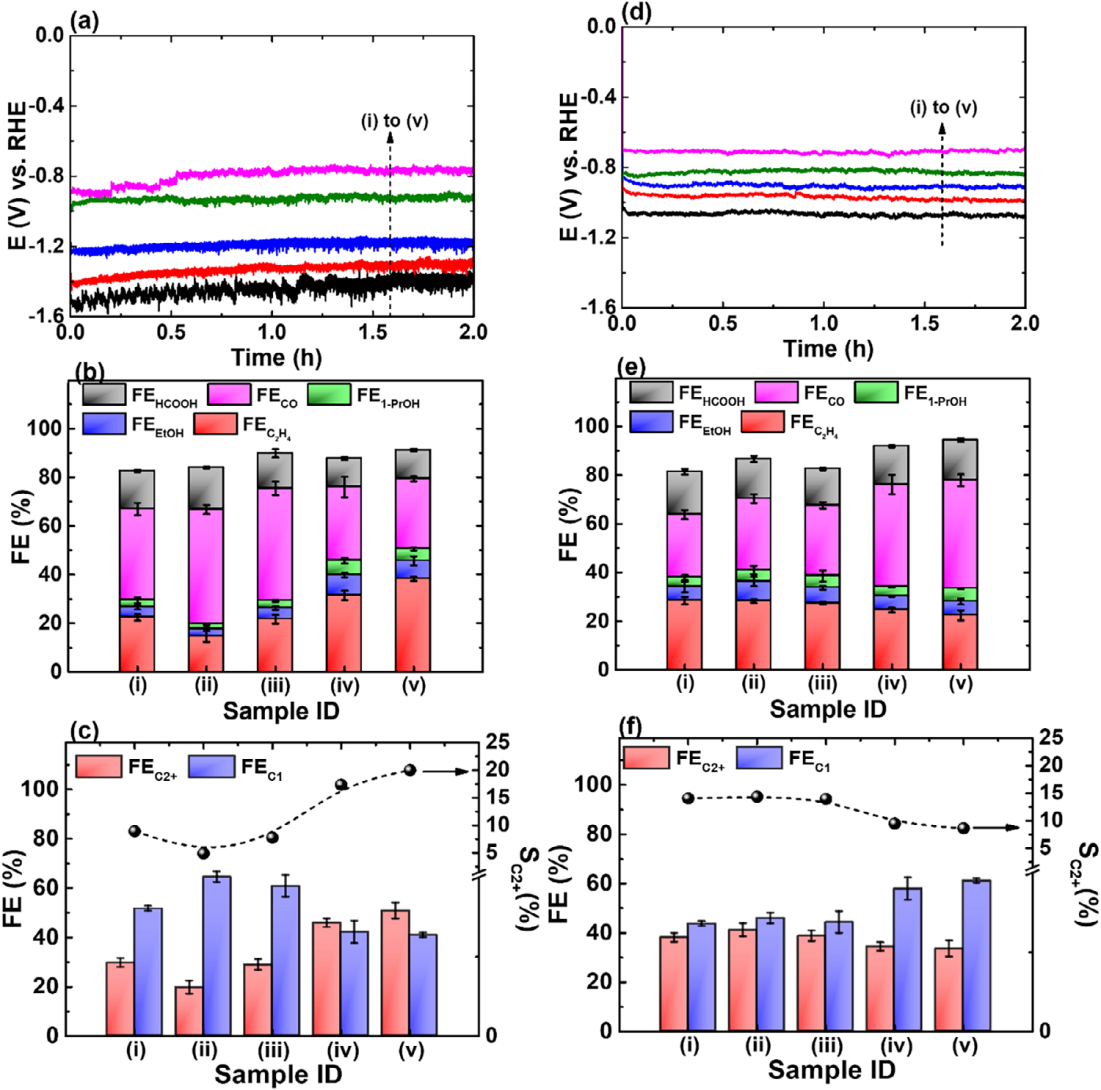
*e*‐CO_2_RR performance of the GDE|Au@Cu_2_O (*n*) electrodes (i: *n* = 1; ii: *n* = 2; iii: *n* = 3; iv: *n* = 4; v: *n* = 5) obtained from 2‐h electrolysis at −200 mA cm^−2^ in (a–c) a blank 1.0 M KHCO_3_ solution and (d–f) a 1.0 M KHCO_3_ solution containing 0.05 M KI: (a,d) potential transients, (b,e) FE for the generated products, and (c,f) summary of FE_C2+_, FE_C1_, and S_C2+_. Error bars represent the standard deviation calculated from at least three independent replicates.

To elucidate the underlying origin of this enhanced performance, control experiments were performed using GDE|Au and GDE|Cu_2_O electrodes under identical operating conditions (Figure S6). SEM and XRD analyses (Figure S7) confirm that the GDE|Au and GDE|Cu_2_O electrodes consisted of crystalline Au and Cu nanoparticles, respectively. Consistent with previous reports [43, 44, 45], the GDE|Au electrode proved incapable of producing C_2+_ products, yielding exclusively C_1_ products (CO and HCOOH) with a minimal total Faradaic efficiency of 5.9 ± 0.9% (Figure S6a). Instead, a pronounced competing HER dominated with a FE_H2_ of 94.2 ± 1.4%. On the other hand, while the product distribution on the GDE|Cu_2_O electrode was qualitatively similar to that of GDE|Au@Cu_2_O (*n*) electrodes, its overall *e*‐CO_2_RR performance was significantly inferior (Figure S6b). Specifically, the formation of C_2+_ products peaked during the first 30 min of electrolysis (14.8 ± 0.9%) but abruptly decayed to 4.5 ± 0.6% after 2‐h electrolysis, accompanied by severe competing HER (FE_H2_: 92.1 ± 1.9%). Collectively, these control experiments confirm that neither component alone can sustain substantial formation of C_2+_ products. At high current densities (e.g., −200 mA cm^−2^), local hydrodynamic factors, gas‐diffusion electrode porosity, and local alkalinization within the catalyst layer are well‐established key factors governing reactant transport and C–C coupling selectivity [46]. Alongside these local microenvironmental phenomena, experimental characterizations indicate that interfacial electron transfer effects between the Au core and Cu_2_O shell serve as a major driver for stabilizing key reaction intermediates and enhancing catalytic durability.

Figure 3 shows post‐electrolysis XPS and AES analyses of the GDE|Au@Cu_2_O (*n* = 2) and GDE|Au@Cu_2_O (*n* = 5) electrodes. As revealed in Figure 3a,e (curve i), both electrodes exhibited two distinct peaks in the Cu 2p_3/2_ region, corresponding to Cu^+^/Cu^0^ and Cu^2+^ species, respectively [11, 47, 48]. Specifically, the GDE|Au@Cu_2_O (*n* = 2) electrode exhibited these features at 931.9 and 933.8 eV, whereas the GDE|Au@Cu_2_O (*n* = 5) electrode displayed corresponding peaks at 932.3 and 934.1 eV. AES analyses (Figure 3b,f, curve i) further distinguished these states, revealing three peaks at 568.0, 570.3, and 573.5 eV, assignable to Cu^0^, Cu^2+^/Cu^+^, and the transition states of the Cu LMM spectrum, respectively [11, 42]. Quantification of surface copper species (sample i in Figure 3d,h) reveals that the post‐electrolysis GDE|Au@Cu_2_O (*n* = 2) electrode consisted of 26.9% Cu^0^, 46.4% Cu^+^, and 26.7% Cu^2+^, corresponding to an average oxidation state (OS_Cu_) of ∼1.00. In contrast, the GDE|Au@Cu_2_O (*n* = 5) electrode contained 50.7% Cu^0^, 22.3% Cu^+^, and 27.0% Cu^2+^, yielding a lower OS_Cu_ of ∼ 0.76. These findings strongly align with the prior literature demonstrating that a moderate OS_Cu_ (typically∼ + 0.5) is highly favorable for promoting C─C coupling [49]. This trend is consistent with the superior FE_C2+_ observed for the GDE|Au@Cu_2_O (*n* = 5) electrode compared to the GDE|Au@Cu_2_O (*n* = 2) electrode.

**FIGURE 3:**
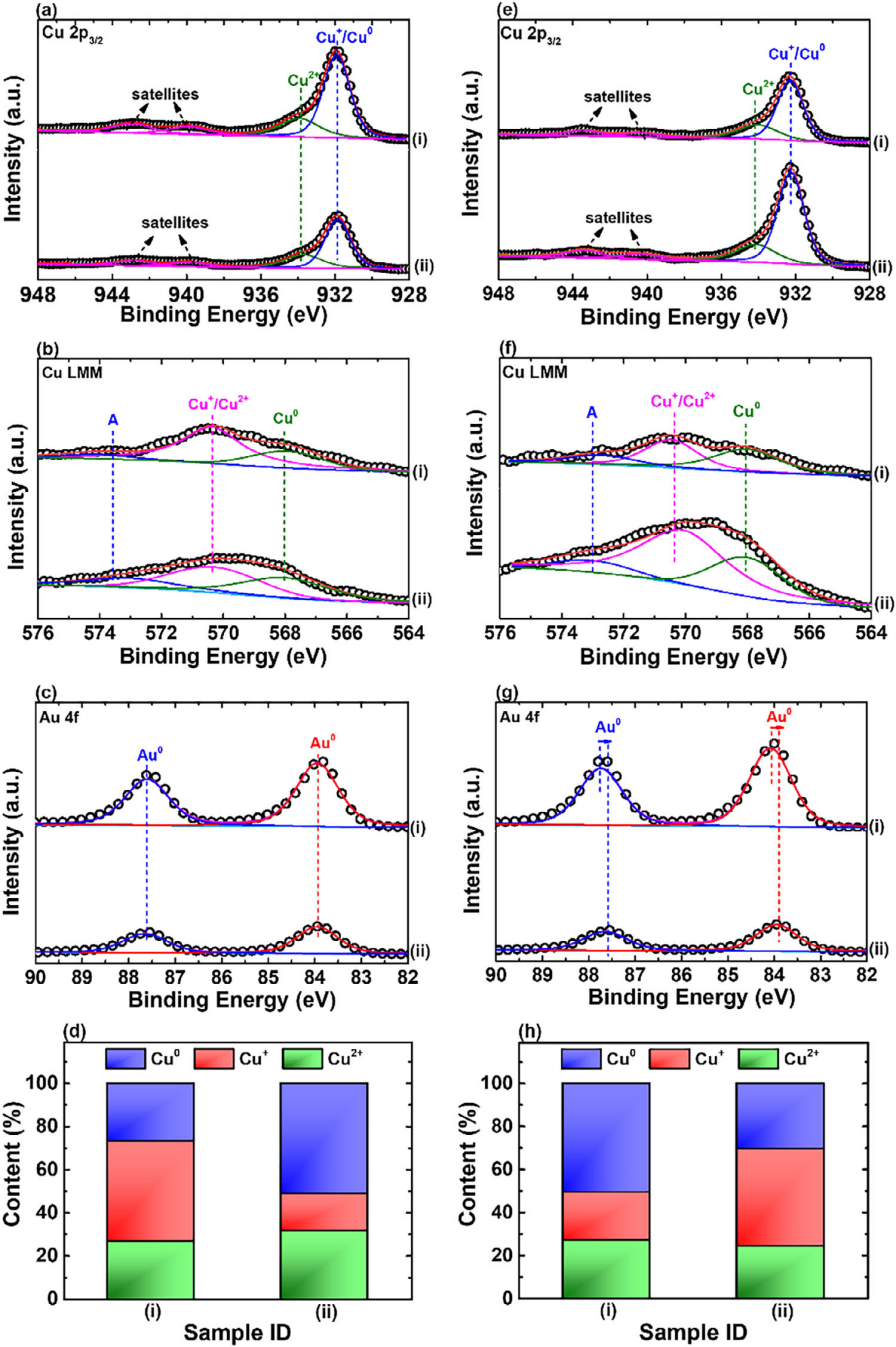
Post‐electrolysis characterization of the (a–d) GDE|Au@Cu_2_O (*n* = 2) and (e–h) GDE|Au@Cu_2_O (*n* = 5) electrodes after 2‐h electrolysis at −200 mA cm^−2^ in (i) a blank 1.0 M KHCO_3_ solution and (ii) a 1.0 M KHCO_3_ solution containing 0.05 M KI: (a,e) high‐resolution Cu 2p XPS spectra, (b,f) Cu LMM Auger spectra, (c,g) Au 4f XPS spectra, and (d,h) relative content of surface copper species quantified from the XPS and AES analyses. Peak A in panels (b) and (f) denotes an auxiliary transition state within the Cu LMM spectrum.

To independently verify the evolution of surface oxidation states and complement the XPS/AES analyses, post‐electrolysis Raman spectroscopy was performed (Figure S8). The results reveal that characteristic vibrational bands associated with oxidized copper phases, including CuO and malachite, are pronounced for the GDE|Au@Cu_2_O (*n* = 2) electrode (curve ii in Figure S8a). In contrast, negligible signals corresponding to malachite are observed for the GDE|Au@Cu_2_O (*n* = 5) electrode (curve ii in Figure S8b). This finding confirms that thinning the Cu_2_O shell effectively suppresses the formation of inactive malachite during high‐rate e‐CO_2_RR. Moreover, post‐electrolysis TEM (Figure S9a–c), HRTEM (Figure 4a,b), and nanobeam electron diffraction (NBED, Figure 4c,d) analyses reveal that the initial core@shell architecture of the Au@Cu_2_O nanoparticles (*n* = 2) underwent a significant morphological reconstruction, transforming into a heterostructure consisting of metallic Au nanoparticles embedded within a CuO_x_ microrod matrix composed of crystalline Cu, CuO, Cu_2_O, and inactive Cu_2_CO_3_(OH)_2_ phases. In contrast, the Au@Cu_2_O (*n* = 5) nanoparticles maintained their core@shell architecture, with a shell composed of Cu, CuO, and Cu_2_O phases, notably without the formation of the inactive Cu_2_CO_3_(OH)_2_ phase (Figure S10a; Figure 4i,j). Collectively, these results indicate that a thinner Cu_2_O shell facilitates pronounced interfacial electronic interactions with the metallic Au core, which plays a multifaceted mechanistic role during *e*‐CO_2_RR. Specifically, interfacial electron transfer between the Au core and Cu_2_O shell regulates OS_Cu_, establishing abundant and stable Cu^0^/Cu^+^ interfaces that simultaneously promote CO_2_ activation and stabilize C─C coupling intermediates [12, 13, 16, 50]. Furthermore, this core–shell electronic coupling stabilizes active reaction sites and preserves the structural integrity of the architecture against phase transformation into inactive malachite, thereby reducing operating overpotentials and substantially enhancing both FE_C2+_and S_C2+_ during electrolysis.

**FIGURE 4:**
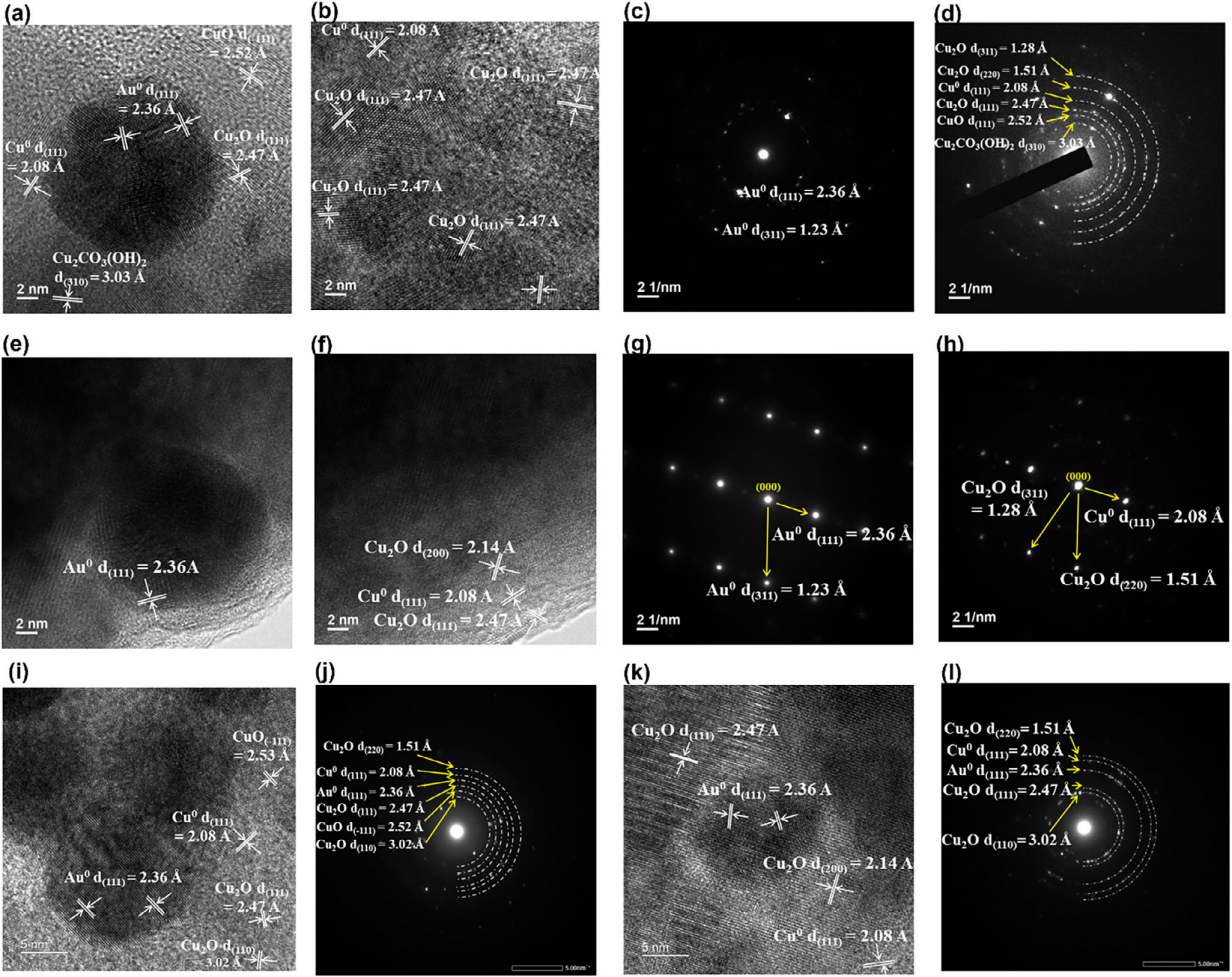
Post‐electrolysis characterization of the (a–h) GDE|Au@Cu_2_O (*n* = 2) and (i–l) GDE|Au@Cu_2_O (*n* = 5) electrodes after 2‐h electrolysis at ‐200 mA cm^−2^ in a blank 1.0 M KHCO_3_ solution (a–d,i,j), and a 1.0 M KHCO_3_ solution containing 0.05 M KI (e‐h and k‐l): (a,b,e,f,i,k) HRTEM images, (c,d,g,h) corresponding NBED patterns, and (j,l) corresponding SAED patterns. (c,g) were obtained from the red‐boxed and green‐boxed regions highlighted in Figure S4c, respectively, whereas (d,h) were obtained from the red‐boxed and green‐boxed regions in Figure S4f, respectively.

Figure 2d–f illustrates the electrochemical characterization of the *e*‐CO_2_RR performance for the GDE|Au@Cu_2_O (*n*) electrodes at ‐200 mA cm^−2^ in 1.0 M KHCO_3_ solution containing 0.05 M KI. As revealed, the response to the iodide electrolyte modification is not an isolated binary switch; rather, it follows a smooth, continuous trend modulated by the Cu_2_O shell thickness. Specifically, the introduction of iodide exerts a progressive effect on product distribution, enhancing the S_C2+_ for the GDE|Au@Cu_2_O (*n*) electrodes with thicker Cu_2_O shells (n ≤3). In the presence of 0.05 M KI, the S_C2+_ values for the GDE|Au@Cu_2_O (*n* = 1), GDE|Au@Cu_2_O (*n* = 2), GDE|Au@Cu_2_O (*n* = 3), GDE|Au@Cu_2_O (*n* = 4), and GDE|Au@Cu_2_O (*n* = 5) electrodes are 14.1%, 14.4%, 13.9%, 9.5%, and 8.6%, respectively, compared to baseline values of 8.9%, 4.8%, 7.8%, 17.4%, and 20.0% in the iodide‐free electrolyte. Notably, the presence of 0.05 M iodide significantly enhanced both FE_C2+_ (40.9 ± 3.2% vs. 19.6 ± 2.7%) and S_C2+_ (14.4% vs. 4.8%) of the thick‐shelled GDE|Au@Cu_2_O (*n* = 2) electrode compared to the values obtained in the iodide‐free electrolyte. In stark contrast, the presence of 0.05 M iodide significantly deteriorated the FE_C2+_ (33.4 ± 3.3% vs. 50.6 ± 3.3%) and S_C2+_ (8.6% vs. 20%) of the thin‐shelled GDE|Au@Cu_2_O (*n* = 5) electrode compared to its performance in the blank 1.0 M KHCO_3_ electrolyte. Control experiments on monometallic GDE|Au and GDE|Cu_2_O electrodes in iodide (0.05 M)‐containing medium (Figure S11) showed that the FE_C1_ of the GDE|Au electrode increased marginally from 5.9 ± 0.9% to 7.2 ± 1.9%. Meanwhile, the presence of iodide enhanced both FE_C1_ and FE_C2+_ of the GDE|Cu_2_O electrode, with its FE_C1_ increasing from 6.9 ± 0.9% to 14.0 ± 2.2% and its FE_C2+_ rising from 4.5 ± 0.6% to 14.7 ± 1.0%. This finding aligns with previous literature demonstrating that the presence of iodide is highly beneficial for improving the FE_C2+_ of copper‐based electrocatalysts [10, 21, 33, 51]. Nevertheless, the overall *e*‐CO_2_RR performance of the GDE|Cu_2_O electrode remains strictly inferior to those of both GDE|Au@Cu_2_O (*n* = 2) and GDE|Au@Cu_2_O (*n* = 5) electrodes, confirming the critical role of the metallic Au core.

Post‐electrolysis XPS (curve ii in Figure 3a,e) and AES analyses (curve ii in Figure 3b,f) confirm that both electrodes after operation in the iodide‐containing electrolyte exhibited features corresponding to Cu^+^, Cu^0^, and Cu^2+^, indicating that the Cu_2_O shell also undergoes a distinct chemical restructuring in the presence of iodide. However, quantified surface compositions (Figure 3d,h, sample ii) revealed a complete inversion of oxidation state trends compared to the blank electrolyte. Following electrolysis with iodide, the GDE|Au@Cu_2_O (*n* = 2) electrode consisted of 51.4% Cu^0^, 17.2% Cu^+^, and 31.4% Cu^2+^, shifting its OS_Cu_ down to ∼0.8. In contrast, the GDE|Au@Cu_2_O (*n* = 5) electrode contained 30.6% Cu^0^, 45.2% Cu^+^, and 24.2% Cu^2+^, shifting toward a higher OS_Cu_ of ∼ 0.94. Furthermore, the I 3d XPS spectra (Figure S12) reveal that both electrodes exhibit two distinct peaks in the I 3d core‐level region, confirming the presence of adsorbed I^−^ species [21]. Specifically, the thick‐shelled GDE|Au@Cu_2_O (*n* = 2) electrode exhibits these spin‐orbit split features (I 3d_5/2_ and I 3d_3/2_) at binding energies of 619.5 and 631.0 eV, respectively. Similarly, the thin‐shelled GDE|Au@Cu_2_O (*n* = 5) electrode displays corresponding peaks at 619.2 and 630.7 eV. This direct surface analysis unambiguously confirms the presence of specifically adsorbed iodide on the copper‐based shell under working conditions, directly substantiating our mechanistic conclusions regarding iodide‐modulated structural reconstruction and electronic interactions. To further elucidate the role of iodide in suppressing oxidative deactivation and modulating surface species, post‐electrolysis Raman spectroscopy was performed (Figure S8). The Raman spectra confirm that introducing 0.05 M KI into the electrolyte drastically reduces the characteristic vibrational bands of oxidized surface species (e.g., CuO) and completely eliminates the formation of inactive malachite on both electrodes (curve iii in Figure S8a,b). This spectroscopic evidence demonstrates that specific iodide adsorption helps stabilize reduced Cu^0^/Cu^+^ species and prevents destructive phase transformations during high‐rate electrolysis. Parallel to these chemical modifications, post‐electrolysis TEM (Figure S4d–f), HRTEM (Figure 4e,f), and NBED (Figure 4g,h) analyses reveal that the initial core@shell architecture of the Au@Cu_2_O nanoparticles (*n* = 2) was reconstructed into metallic Au nanoparticles‐coated CuO_x_ nanocubes composed of crystalline Cu and Cu_2_O phases. Meanwhile, the Au@Cu_2_O nanocatalysts maintained their core@shell architecture, with a shell composed of crystalline Cu and Cu_2_O phases (Figure S10b; Figure 4k,l). Collectively, these findings demonstrate that iodide modulates the surface chemistry and reconstruction pathways as a function of the shell thickness of Au@Cu_2_O nanocatalyst. For the thick‐shelled Au@Cu_2_O (*n* = 2), iodide facilitates a structural transformation into active nanocubes and lowers the OS_Cu_ toward a more favorable regime, significantly boosting *e*‐CO_2_RR performance. Conversely, for the thin‐shelled Au@Cu_2_O (*n* = 5), the strong adsorption or interaction of iodide disrupts the pre‐existing favorable core@shell electronic modulation, driving the OS_Cu_ away from the highly favorable regime and suppressing S_C2+_.

Figure 5 illustrates the electrochemical characterization of the *e*‐CO_2_RR performance for the GDE|Au@Cu_2_O (*n* = 2) and GDE|Au@Cu_2_O (*n* = 5) electrodes at ‐200 mA cm^−2^ in 1.0 M KHCO_3_ solution containing various concentrations of KI. As revealed, the introduction of iodide exerted a distinctly divergent effect on the product distribution between the two electrodes. Specifically, the introduction of iodide deteriorated the S_C2+_ of the GDE|Au@Cu_2_O (*n* = 5) electrode across the entire iodide concentration range of interest (0.01 M ∼ 0.1 M) (Figure 5d). In contrast, the presence of low concentrations (0.01 M∼ 0.05 M) of iodide significantly enhanced the S_C2+_ of the GDE|Au@Cu_2_O (*n* = 2) electrode, with its S_C2+_ reaching a maximum when the electrolysis was performed with 0.05 M KI (Figure 5a). On the other hand, the presence of iodide is beneficial to reduce the operating potentials (E_op_) of both electrodes required to sustain the applied current density of ‐200 mA cm^−2^ (Figure 5b,e). For instance, the operating potential of the GDE|Au@Cu_2_O (*n* = 2) electrode to maintain the applied current density of −200 mA cm^−2^ during the 2‐h electrolysis in the KI (0.10 M)‐containing KHCO_3_ solution was about 0.57 V less than that in the blank 1.0 M KHCO_3_ solution. In contrast, this depolarization effect was less pronounced for the GDE|Au@Cu_2_O (*n* = 5) electrode, which exhibited only a 0.14 V decrease under identical operating conditions. As evidenced by EIS analyses (Figure S5b and Table S1), the reduced overpotential in the presence of iodide ions stems directly from a significantly lowered R_ct_. Notably, the fitted R_ct_ values of all GDE|Au@Cu_2_O (*n*) electrodes in the presence of iodide ions are lower than those obtained in the absence of iodide. These findings provide direct quantitative evidence that iodide electrolyte engineering substantially enhances interfacial charge‐transfer kinetics during high‐rate *e*‐CO_2_RR. Similar behavior has been observed for the GDE|Au and GDE|Cu_2_O control samples, with potential reduction by 0.2 V and 0.1 V for the GDE|Au and GDE|Cu_2_O electrodes, respectively, upon the introduction of 0.05 M KI (Figures S6 and S11). These results further highlight the role of iodide adsorption in improving the charge‐transfer kinetics and lowering the barrier for CO_2_ activation [36, 52, 53]. Consequently, as summarized in Figure 5c,f, the optimal EE_total_ was achieved at 0.05 M KI, reaching maximum values of 46.9 ± 1.9% and 54.7 ± 2.1% for the GDE|Au@Cu_2_O (*n* = 2) and GDE|Au@Cu_2_O (*n* = 5) electrodes, respectively. Note that the calculated values of EE_total_ represent conservative lower bounds due to the potential overestimation of E_op_ resulting from higher local interfacial pH relative to the bulk pH during high‐rate (i.e., ‐200 mA cm^−2^) electrolysis. Collectively, these results demonstrate that the influence of I^−^ ions on catalytic activity varies dynamically with Cu_2_O shell thickness and catalyst configuration, underscoring the key role of iodide‐promoted surface reconstruction and local reaction microenvironment in governing product selectivity and EE_total_ for the *e*‐CO_2_RR process.

**FIGURE 5:**
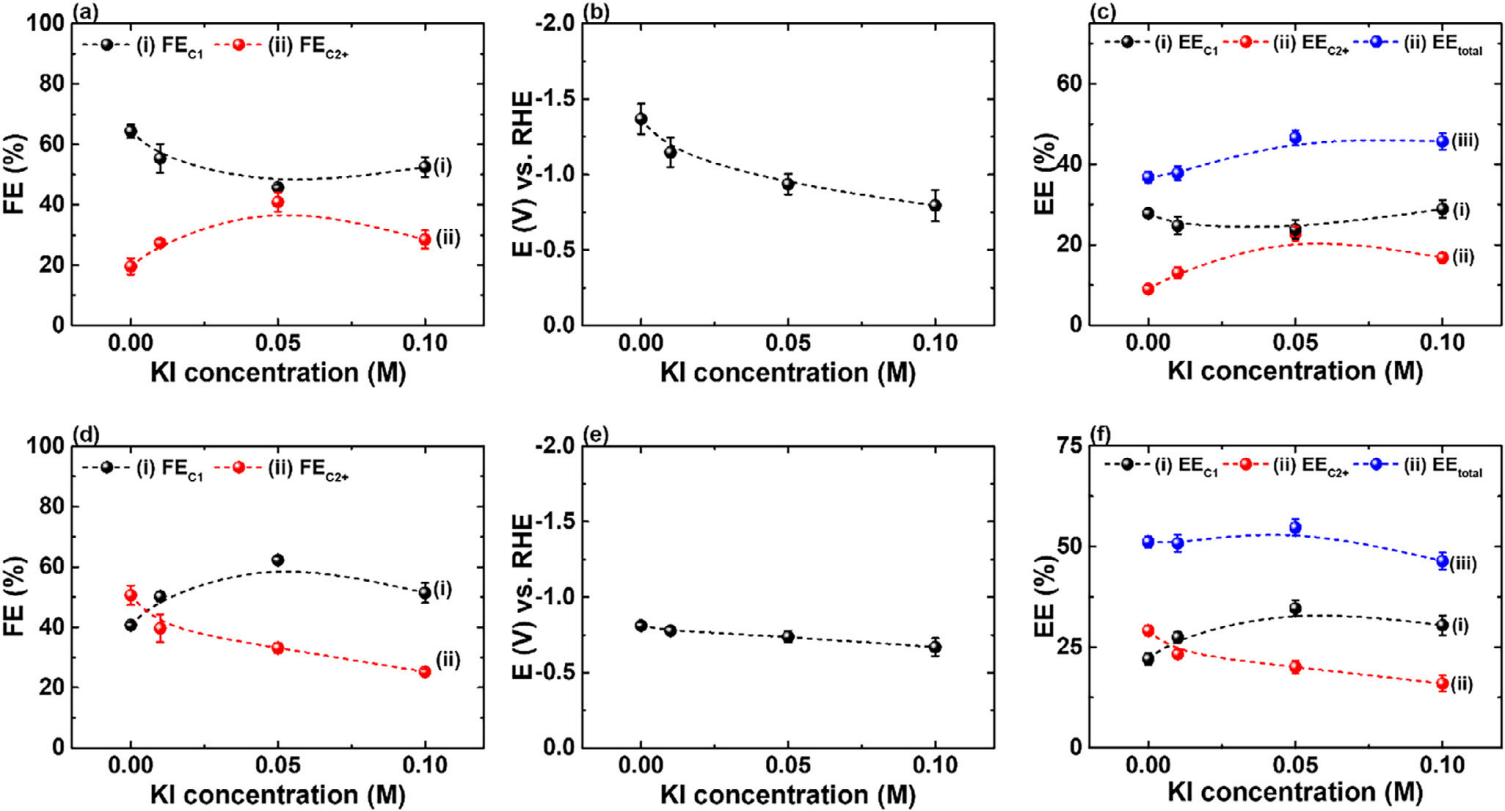
*e*‐CO_2_RR performance of the (a–c) GDE|Au@Cu_2_O (*n* = 2) and (d–f) GDE|Au@Cu_2_O (*n* = 5) electrodes obtained from 2‐h electrolysis at −200 mA cm^−2^ in the 1.0 M KHCO_3_ solution containing various concentration of KI: (a,d) the FE for the generated products, (b,e) operating potential transients, and (c,f) a summary of EE_C1_, EE_C2+_, and EE_total_. Error bars represent the standard deviation calculated from at least three independent replicates.

Interestingly, the EE_total_ of the GDE|Au@Cu_2_O (*n* = 5) electrode in the blank 1.0 M KHCO_3_ solution was 51.2 ± 1.4%, which is close to that obtained in the presence of 0.05 M KI. Furthermore, following an extended 24‐h electrolysis (Figure S13), the GDE|Au@Cu_2_O (*n* = 5) electrode in the blank 1.0 M KHCO_3_ solution firmly retained its high *e*‐CO_2_RR performance, maintaining a FE_C2+_ of 38.0% and an EE_C2+_ of 22.4% (EE_total_: 46.5%). Crucially, rigorous post‐electrolysis characterizations confirm the structural and chemical integrity of the catalyst after this prolonged operation. Post‐electrolysis SEM and TEM analyses (Figure S14) confirm that the Au@Cu_2_O core@shell architecture is well‐preserved. Additionally, post‐electrolysis Raman analyses (Figure S15) reveal that an absence of vibrational bands corresponding to inactive malachite, confirming that this destructive phase transformation was successfully suppressed over the 24‐h period. Quantitative post‐electrolysis XPS and AES surface deconvolutions (Figure S16) reveal that the extended‐operation GDE|Au@Cu_2_O (*n* = 5) electrode consisted of 29.6% Cu^0^, 65.7% Cu^+^, and 4.7% Cu^2+^, corresponding to an average oxidation state (OS_Cu_) of ∼0.75. This outstanding durability and efficiency highlight that the enhanced selectivity and stability uniquely arise from favorable electron transfer effects at the Au@Cu_2_O core@shell interface. Ultimately, the excellent *e*‐CO_2_RR performance of the GDE|Au@Cu_2_O (*n* = 5) electrode, in terms of the high FE_C2+_ at a low operating potential under an industrially relevant current density of ‐200 mA cm^−2^, places it as the most active Au‐Cu bimetallic Cu‐based electrocatalyst at near‐neutral conditions (Table S2).

## 3 Conclusion

In summary, we have demonstrated that the *e*‐CO_2_RR selectivity, stability, and overpotential of bimetallic Au@Cu_2_O core@shell catalysts can be precisely tailored by modulating the Cu_2_O shell thickness and exploiting iodide‐mediated surface reconstructions. Our findings reveal that the Au@Cu_2_O (*n* = 5) catalyst, featuring a thin shell of ∼ 11.4 nm, exhibits superior intrinsic performance in a blank 1.0 M KHCO_3_ electrolyte under an industrially relevant current density of ‐200 mA cm^−2^, delivering a high FE_C2+_ of 50.6 ± 3.2% and EE_total_ of 51.2 ± 1.4%. This superior performance stems from a profound interfacial electronic interaction that optimizes the average surface copper oxidation state (OS_Cu_: ∼ +0.76) and preserves the structural integrity of the core@shell architecture, effectively preventing degradation into the inactive malachite phase (Cu_2_CO_3_(OH)_2_). Conversely, the thicker shell (19.0 ± 2.0 nm) of the Au@Cu_2_O (*n* = 2) variant leads to an unfavorable OS_Cu_ of ∼1.00, causing the Cu_2_O shell to reconstruct into a microrod matrix containing the inactive copper carbonate phase. Intriguingly, the introduction of iodide ions completely inverts these catalytic pathways. For the thicker‐shelled Au@Cu_2_O (*n* = 2) nanocatalyst, iodide adsorption drives a beneficial, constructive reconstruction of Au@CuO_x_ nanocubes while shifting the OS_Cu_ toward a more favorable regime (+0.80), thereby dramatically boosting S_C2+_ and suppressing overpotentials. In sharp contrast, for the thin‐shelled Au@Cu_2_O (*n* = 5) nanocatalyst, strong iodide adsorption disrupts the pre‐existing, highly favorable core@shell electron transfer effect, driving the OS_Cu_ away from the favorable regime to ∼+0.94 and thereby suppressing the production of C_2+_ products. Nevertheless, iodide universally functions as an efficient interfacial kinetic promoter that substantially mitigates charge‐transfer resistance, lowering operating potentials across all configurations and yielding an exceptional peak EE_total_ of 54.7 ± 2.1% for the GDE|Au@Cu_2_O (*n* = 5) system. Furthermore, this optimal thin‐shelled catalyst demonstrates exceptional long‐term stability, maintaining a robust FE_C2+_ of 38.0% and completely preserving its core–shell structural integrity without inactive malachite formation over 24 h of continuous high‐rate electrolysis. Coupled with its outstanding operational durability, the GDE|Au@Cu_2_O (*n* = 5) electrode stands out as a state‐of‐the‐art AuCu bimetallic electrocatalyst in near‐neutral media at high current densities. Beyond this specific material system, this work offers a sophisticated, transferable blueprint for the future development of high‐rate gas‐diffusion electrocatalytic systems. It underscores a vital paradigm shift for the field: achieving maximum efficiency in practical carbon mitigation requires the synchronized, cooperative engineering of both interfacial electronic structures and local electrolyte microenvironments.

## 4 Experimental Section/Methods

### 4.1 Chemical and Materials

All the chemicals, including potassium iodide potassium hydroxide (≥85%, Honeywell Fluka), (≥99.5%, Honeywell Fluka), potassium bicarbonate (99%, Alfa Aesar), potassium chloride (≥99.0%, Sigma–Aldrich), sodium hydroxide (≥98%, Honeywell Fluka), hydrochloric acid (≥37% Honeywell Fluka), and hydrogen peroxide solution (30%, Honeywell Fluka), were used as received from commercial suppliers without further purification. Deionized water (18.2 MΩ•cm; DIW) was used for the electrode rinsing and electrolyte preparation throughout the work. Nickel foam (1.0 mm thickness, >99%) and GDL‐340 carbon paper (340 µm thickness, featuring a microporous layer) were purchased from Central Research Technology Co. (Taiwan) and CeTech Co., Ltd. (Taiwan), respectively. Taiwan. Prior to use, the Ni foam was cleaned by immersion in 1.0 M HCl solution for 5 min to remove surface oxides, followed by thorough rinsing with DIW.

### 4.2 Catalyst Synthesis and Electrode Fabrication

Au@Cu_2_O core@shell nanoparticles were prepared according to previously reported procedures [38]. Briefly, Au core seed solution (0.25 mM) was prepared using a standard citrate reduction method. To synthesize Au@Cu_2_O core@shell nanoparticles with varying thicknesses (designated as Au@Cu_2_O (*n* = 1–5)), specific volumes (1.0, 2.0, 3.0, 4.0, and 5.0 mL) of Au core seed solutions (0.25 mM) were slowly introduced into a two‐neck round‐bottom flask containing 15 mL CuSO_4_ aqueous solution (1.33 mM), followed by the injection of 21.5 mL of an aqueous solution containing NaOH (0.07 M) and ascorbic acid (2.33 mM). After stirring for 10 min at 35°C, the Au@Cu_2_O (*n* = 1−5) nanoparticles were collected by centrifugation and dried overnight at 25°C in a vacuum oven. Control samples of pristine Cu_2_O nanocrystals were prepared under identical conditions without Au, while pristine Au nanoparticles were obtained by drying the centrifuged Au core seed solution.

The prepared powders were subsequently dispersed in ethanol (2.4 mg mL^−1^) under continuous ultrasonication for 2 h to form the catalyst inks. Prior to electrode fabrication, the GDL‐340 carbon paper was cleaned by sequential ultrasonication in hydrochloric acid (5 min), ethanol (5 min), and deionized water (10 min). Finally, the prepared catalyst inks (0.2 mL cm^−2^) were drop‐casted on the cleaned GDL‐340 substrates. For simplicity, the resultant electrodes were designed as GDE|Au@Cu_2_O (*n*), GDE|Cu_2_O, and GDE|Au.

### 4.3 Physical Characterization

The surface morphology and chemical composition of prepared samples were characterized using scanning electron microscopy (SEM, Hitachi SU‐8010) equipped with energy‐dispersive X‐ray spectroscopy (EDS). X‐ray diffraction (XRD) was performed using a Bruker D8 DISCOVER X‐ray diffractometer. X‐ray photoelectron spectroscopy (XPS) and Auger Spectroscopy (AES) were performed using a PHI 5000 VersaProbe system (ULVAC‐PHI, Chigasaki, Japan). All XPS spectra were calibrated by referencing the C 1s peak to a binding energy of 284.8 eV. Detailed structural and elemental analyses were performed by TEM and HRTEM with a JEM‐2100F microscope (JEOL Ltd., Japan). The structural evolution and surface phases of the prepared electrodes were characterized by Raman spectroscopy (Protrustech Corporation Ltd.) equipped with a 532 nm excitation laser.

### 4.4 Electrochemical Characterization

Electrochemical measurements were performed using an IviumNStat multi‐channel potentiostat (Ivium Technologies B.V., Netherlands). A customized three‐electrode two‐compartment flow‐type electrolyzer (Scheme S1) was employed, with the anodic and cathodic compartments separated by a Neosepta ASE anion exchange membrane (ASTOM Corporation, Tokyo, Japan). The prepared electrodes were used as the working electrode, while nickel foam (3.5 cm × 3.5 cm) and Ag/AgCl (sat'd KCl) were employed as the counter electrode and reference electrode, respectively. High‐purity CO_2_ gas (98% CO_2_ and 2% CH_4_; Yun Shan Gas Co., Taiwan) was delivered to the cathodic compartment at a constant flow rate (*ϕ*) of 35 mL min^−1^, regulated by a mass flow controller throughout the reaction process. 2% CH_4_ served as the internal standard for the quantification of gaseous products via gas chromatography (GC). The catholyte and anolyte consist of KHCO_3_ (1.0 M) and KOH (1.0 M) solutions, respectively, both of which were circulated through the cathodic and anodic compartments of the electrolyzer at 35 mL min^−1^ using peristaltic pumps. Potentials recorded during constant‐current electrolysis were manually corrected with 95% iR compensation. All potentials reported in this work are referenced to the reversible hydrogen electrode (RHE) using Equation (1):

(1) $$E\left(V\;vs.\;\mathrm{RHE}\right)=E\;\left(V\mathrm{vs}.\;\mathrm{Ag}/\mathrm{AgCl}\right)+0.197+0.059\times \mathrm{pH}$$

It should be noted that reported potentials are calculated based on bulk pH. Because rapid OH^−^ generation during electrolysis at high applied current density (i.e., −200 mA cm^−2^) yields a local interfacial pH considerably higher than the bulk value, the true operating potentials vs RHE may be overestimated.

Electrochemical impedance spectroscopy (EIS) measurements of all GDE|Au@Cu_2_O (*n*) electrodes were performed in both blank 1.0 M KHCO_3_ and 0.05 M KI‐containing 1.0 M KHCO_3_ electrolyte solutions using an IviumNStat multi‐channel potentiostat (Ivium Technologies B.V., Netherlands) equipped with an EIS spectrum analyzer. The spectra were recorded by applying an AC amplitude of 10 mV over a frequency range from 1 MHz to 0.1 Hz at the open‐circuit potential. To quantitatively deconvolute the kinetic contributions, the EIS spectra were fitted using an equivalent circuit consisting of series resistance (R_s_) with two series‐connected RC circuits [54, 55]. The first RC circuit, consisting of a charge‐transport resistance (*R*_t_) and a constant phase element related to the double‐layer capacitance, contributes to the impedance at higher frequencies. The other RC circuit, consisting of an interfacial charge‐transfer resistance at the interface of the electrode/electrolyte (R_ct_) and a constant phase element related to the bulk Faradaic pseudocapacitance, contributes to the impedance at lower frequencies.

### 4.5 Product Analysis and Quantification

The liquid products, including ethanol and 1‐propanol, generated during the constant‐current electrolysis were analyzed and quantified through GC (Shimadzu GC‐2010 Plus). Formate was quantified at the conclusion of each electrolysis using ion chromatography (IC; Metrohm 883 Basic IC) equipped with Metrosep Organic Acids (7.8 * 250 mm) and Metrosep Organic Acids Guard (4.6 * 50 mm). The GC was equipped with an Rtx‐Volatiles column and a flame ionization detector (FID). The oven temperature was initially held at 40°C for 1 min, followed by a ramp of 40°C min^−1^ to 80°C (held for 6 min), and a final ramp of 20°C min^−1^ to 250°C (held for 8.5 min).  Gaseous products, on the other hand, were analyzed and quantified using an Agilent 7890B Series gas chromatography system. CO and ethylene were quantified using a Carboxen‐1006 PLOT column with helium carrier gas (∼5 mL min^−1^), coupled to a thermal conductivity detector (TCD) and a FID connected in series. Hydrogen gas was analyzed using an HP‐PLOT Molesieve 19095P‐MS6 column with nitrogen carrier gas (∼4 mL min^−1^), coupled to a TCD detector. The oven temperature was initially maintained at 40°C for 8 min, then ramped at 40°C min^−1^ to 200°C and held at 200°C for 8 min. Product concentrations (C_product_) were determined from IC and GC signals using the routinely updated calibration curves. The generation rate (R_product_) the gas products and liquid products were determined using Equations (2) and (3), respectively. The selectivity for C_2+_ products (S_C2+_) was determined by dividing the generation rates of C_2+_ products (R_C2+_) by the combined generation rates of C_1_ (R_C1_) and C_2+_ products. It should be noted that because H_2_ originates from the competing HER rather than *e*‐CO_2_RR, S_C2+_ evaluates the intrinsic catalytic capability for C─C coupling among transformed carbonaceous species, whereas overall current utilization across all reactions (including HER) is evaluated using Faradaic efficiency (*vide infra*).

(2) $$R_{\mathrm{product}}=\phi \times C_{\mathrm{product}}\times \frac{P}{\mathrm{RT}}$$

(3) $$R_{\mathrm{product}}=\frac{V_{\mathrm{catholyte}}\times C_{\mathrm{product}}}{t}$$

where P is the ambient pressure (1 atm), R is the ideal gas constant (0.082 L atm K^−1^ mol^−1^), T is the operating temperature (298.15 K), V_catholyte_ is the volume of the catholyte, and t is the duration of electrolysis.

The Faradaic efficiency (FE_product_) for a specific product was determined using Equation (4)

(4) $$FE_{\mathrm{product}}=\frac{R_{\mathrm{product}}\times n_{\mathrm{product}}\times F}{J}\times 100\%$$

where n_product_ is the number of electrons transferred for the formation of the specific product, F is the Faradaic constant (96,485 C mol^−1^), and J is the applied current density during the reaction.

The cathodic energy efficiencies for C_1_ (EE_C1_) and C_2+_ (EE_C2+_) products were determined as the ratio of chemical energy stored in the generated C_1_ and C_2+_ products to the applied electrical energy using Equation (5) [10]:

(5) $$EE_{P}=\frac{\sum_{i}\left(1.23-E_{i}^{0}\right)\times FE_{i}}{1.23-E_{op}}\times 100\%\left(P:C_{1}\mathrm{and}C_{2+}\right)$$

where E_i_^0^, FE_i_, and E_op_ denote the standard reduction potential of a specific product i, its corresponding Faradaic efficiency, and the operating potential under the applied current density, respectively. The total cathodic energy efficiency (EE_total_) for all generated *e*‐CO_2_RR products is thus defined as the sum of EE_C1_ and EE_C2+_. Note that the calculated values of EE_C1_, EE_C2+_, EE_total_ represent conservative lower bounds due to the potential overestimation of E_op_ resulting from higher local interfacial pH relative to the bulk pH during high‐rate (i.e., −200 mA cm^−2^) electrolysis.

### 4.6 Statistical Analysis

The data was reported as mean value ± standard deviation. The reported standard deviation was calculated from a minimum of three independent repeated experiments.

## Author Contributions

**Eunice Estrella De Guzman**: investigation, methodology, writing – original draft, formal analysis, data curation, validation. **Jen‐An Shih**: investigation, methodology, validation, formal analysis, data curation. **Wan‐Ru Lee**: investigation. **Yung‐Jung Hsu**: funding acquisition, writing – original draft, writing – review and editing, conceptualization, supervision. **Chia‐Yu Lin**: funding acquisition, writing – review and editing, writing – original draft, conceptualization, supervision.

## Conflicts of Interest

The authors declare no conflicts of interest.

## Supporting information




**Supporting File**: smll76442‐sup‐0001‐SuppMat.docx.

## Data Availability

The data that support the findings of this study are available from the corresponding author upon reasonable request.
