# Supplementary material for: Cooperative Interfacial and Electrolyte Engineering in Au@Cu2O Core@Shell Nanocatalysts for Efficient CO2 Reduction to C2+ Products at Circumneutral pH
Source: Small. 2026 Oct 24;22(63):e76442. doi: 10.1002/smll.76442 (PMC13755994; doi:10.1002/smll.76442)
Supplement: Supplementary file 1 [file SMLL-22-e76442-s001.docx]

Electronic Supplementary Information

**Cooperative Interfacial and Electrolyte Engineering in Au@Cu_2_O Core@Shell Nanocatalysts for Efficient CO_2_ Reduction to C_2+_ Products at Circumneutral pH**

*Eunice Estrella De Guzman^a^*^‡^*, Jen-An Shih^b^*^‡^*, Wan-Ru Lee^b^, Yung-Jung Hsu^b,c,d*^, Chia-Yu Lin^a,e,f*^*

^a^Department of Chemical Engineering, National Cheng Kung University, Tainan City 70101, Taiwan

^b^Department of Materials Science and Engineering, National Yang Ming Chiao Tung University, Hsinchu 300093, Taiwan

^c^Center for Emergent Functional Matter Science, National Yang Ming Chiao Tung University, Hsinchu 300093, Taiwan

^d^Institute of Integrated Research, Institute of Science Tokyo, Kanagawa 226-8503, Japan

^e^Center for Resilience and Intelligence on Sustainable Energy Research (RiSER), National Cheng Kung University, Tainan 70101, Taiwan

^f^Department of Chemical Engineering, Faculty of Engineering, Chulalongkorn University, Bangkok 10330, Thailand

^‡^These authors equally contributed to the work.

***Corresponding author:**cyl44@mail.ncku.edu.tw (C.Y. Lin) and [yhsu@cc.nctu.edu.tw](mailto:yhsu@cc.nctu.edu.tw) (Y.-J. Hsu)

**Contents**

| Supporting Tables S1-S2 | page S2 |
| --- | --- |
| Supporting Scheme S1 | page S4 |
| Supporting Figures S1-S16 | page S5 |
| References | page S15 |

**Table S1**: Summary of the fitted parameters derived from the equivalent circuit modelling of the electrochemical impedance spectra for the GDE|Au@Cu_2_O (*n*) electrodes in blank 1.0 M KHCO_3_ and 0.05 M KI-containing 1.0 M KHCO_3_ solutions.

|  | R_s_ (Ω) | R_t_ (Ω) | R_ct_ (Ω) |
| --- | --- | --- | --- |
| Electrolyte: 1.0 M KHCO_3_ | |  |  |
| GDE\|Au@Cu_2_O (*n*=1) | 7.5 | 9200 | 6635 |
| GDE\|Au@Cu_2_O (*n*=2) | 7.7 | 9000 | 4900 |
| GDE\|Au@Cu_2_O (*n*=3) | 5.4 | 6900 | 4000 |
| GDE\|Au@Cu_2_O (*n*=4) | 5.5 | 4300 | 1850 |
| GDE\|Au@Cu_2_O (*n*=5) | 4.2 | 1700 | 1200 |
| Electrolyte: 1.0 M KHCO_3_ containing 0.05 M KI | |  |  |
| GDE\|Au@Cu_2_O (*n*=1) | 4.1 | 3900 | 4500 |
| GDE\|Au@Cu_2_O (*n*=2) | 4.1 | 3400 | 3800 |
| GDE\|Au@Cu_2_O (*n*=3) | 5.1 | 1700 | 1700 |
| GDE\|Au@Cu_2_O (*n*=4) | 5.0 | 1500 | 1410 |
| GDE\|Au@Cu_2_O (*n*=5) | 4.8 | 300 | 80 |

**Table S2** Comparison of the e-CO_2_RR performance of the developed GDE|Au@Cu_2_O (*n*=5) electrode at neutral pH with previously reported state-of-the-art Au-Cu bimetallic electrocatalysts.

| **Catalysts** | **Electrolyte** | **Electrolysis condition** | **FE_C2+_ (%)** | **FE_C1_ (%)** | **Ref** |
| --- | --- | --- | --- | --- | --- |
| Au@Cu_2_O (core@shell) | 0.1 M KHCO_3_ | -0.9 V vs. RHE  (~ -15 mA cm^−2^) | ~20 | ~65 | ^1^ |
| Au@Cu_2_O (dumbbell) | 0.1 M KHCO_3_ | -0.9 V vs. RHE  (~ -15 mA cm^−2^) | ~30 | ~55 | ^1^ |
| Au nanoparticles decorated Cu_2_O nanocubes | 0.1 M KHCO_3_ | -1.07 V vs. RHE  (~ -12 mA cm^−2^) | 25 | ~57 | ^2^ |
| Au@Cu_2_O-S-10 yolk-shell nanoreactor | 0.1 M KHCO_3_ | -1.22 V vs. RHE  (~ -20 mA cm^−2^) | ~50 | ~34 | ^3^ |
| Au@Cu_2_O (flower) | 0.1 M KHCO_3_ | -1.0 V vs. RHE  (~ -5 mA cm^−2^) | 0 | ~73 | ^4^ |
| Cu_2_O@Au (hollow cube) | 0.1 M KHCO_3_ | -1.0 V vs. RHE  (~ -5 mA cm^−2^) | ~5 | ~30 | ^5^ |
| AuCu Alloy Nanoparticle Embedded Cu Submicrocone Array | 0.5 M KHCO_3_ | -0.8 V vs. RHE  (-4.9 mA cm^−2^) | ~30 | Not available | ^6^ |
| Laser ablation-controlled Au/Cu structures (Au_80_/Cu \| L=100%) | 0.1 M KHCO_3_ | -1.4 V vs. RHE  (~ -6 mA cm^-2^) | ~36 | ~7 | ^7^ |
| Au nanoparticle anchored on Cu nanocubes with (100) facet | 0.1 M KHCO_3_ | -1.2 V vs. RHE  (~ -6.5 mA cm^-2^) | ~41 | ~29 | ^8^ |
| Compositional gradient Au-Cu heterostructure | 0.1 M KHCO_3_ | -0.4 V vs. RHE  (~ -0.1 mA cm^-2^) | 85 | ~14 | ^9^ |
| Au-Cu nanourchins with low-coordinated Au/Cu atoms | 0.1M KHCO_3_ | -0.4 V vs. RHE  (~-0.17 mA cm^-2^) | ~100 | 0 | ^10^ |
| GDE\|Au@Cu_2_O (*n*=5) | 1 M KHCO_3_ | -200 mA cm^−2^  (~ -0.81 V vs. RHE) | 50.6 ± 3.3 | 40.7 ± 0.9 | This work  This work |
| GDE\|Au@Cu_2_O (*n*=5) | 1M KHCO_3_ + 0.05M KI | -200 mA cm^−2^  (~ -0.74 V vs. RHE) | 33.1 ± 1.7 | 62.3 ± 1.1 |  |

**Scheme S1** Schematic illustration of the developed three-electrode two-compartment flow-type electrolyzer.

**Figure S1** (a) XRD patterns of the as-prepared GDE|Au@Cu_2_O (*n*) electrodes (i: *n*= 1; ii: *n*= 2; iii: *n*= 3; iv: *n*= 4; v: *n*= 5) with peak assignments for Cu_2_O (JCPDS no. 05-0667), Au^0^ (JCPDS no. 04-0784), and C (JCPDS no. 26-1076). (b-f) SEM images of the as-prepared GDE|Au@Cu_2_O (*n*) electrodes (b: *n*= 1; c: *n*= 2; d: *n*= 3; e: *n*= 4; f: *n*= 5).

**Figure S2** (a-e) Low-magnification SEM images and (a’-e’) corresponding particle size distribution histograms of the as-prepared GDE|Au@Cu_2_O (*n*) electrodes (a, a’: *n*= 1; b, b’: *n*= 2; c, c’: *n*= 3; d, d’: *n*= 4; e, e’: *n*= 5).

**Figure S3** High-resolution XPS spectra of (a-d) Au 4f and (a’-d’) Cu 2p_3/2_ core-level regions for the Au@Cu_2_O (*n*=2) electrode obtained after Ar^+^ ion etching for (a, a’) 0 s, (b, b’) 3 s, (c, c’) 5s, and (d, d’) 10s.

**Figure S4** (a) Cu LMM Auger spectra and (b) relative content of surface copper species quantified from combined XPS and AES deconvolution for the as-prepared GDE|Au@Cu_2_O (*n*) electrodes (i: n=2; ii: n=5). Peak A in panels (a) denotes an auxiliary transition state within the Cu LMM spectrum.

**Figure S5** (a-b) Nyquist plots of the GDE|Au@Cu_2_O (*n*) electrodes (i: *n*= 1; ii: *n*= 2; iii: *n*= 3; iv: *n*= 4; v: *n*= 5) recorded in (a) blank 1.0 M KHCO_3_ and (b) 0.05 M KI-containing 1.0 M KHCO_3_ solutions. (c) Equivalent circuit used for impedance parameter fitting.

**Figure S6** e-CO_2_RR performance (FE for the generated products and operating potential) of the (a) GDE|Au and (b) GDE|Cu_2_O electrodes, obtained from 2-h electrolysis at -200 mA cm^−2^ in a blank 1.0 M KHCO_3_ solution.

**Figure S7** (a-b) SEM images of the (a) GDE|Cu_2_O and (b) GDE|Au electrodes. (c) XRD patterns of the GDE|Cu_2_O and GDE|Au electrodes.

**Figure S8** Raman spectra of (a) GDE|Au@Cu_2_O (*n*=2) and (b) GDE|Au@Cu_2_O (*n*=5) electrodes: (i) as-prepared, (ii) following 2-h electrolysis at -200 mA cm^−2^ in the blank 1.0 M KHCO_3_ solution, and (iii) following 2-h electrolysis at -200 mA cm^−2^ in the 1.0 M KHCO_3_ solution containing 0.05 M KI.

**Figure S9** Post-electrolysis TEM images at various magnifications of the GDE|Au@Cu_2_O (*n*=2) electrode after 2-h electrolysis at -100 mA cm^−2^ in (a-c) a blank 1.0 M KHCO_3_ solution and (d-f) a 1.0 M KHCO_3_ solution containing 0.05 M KI.

**Figure S10** Post-electrolysis TEM images of the GDE|Au@Cu_2_O (*n*=5) electrode after 2-h electrolysis at -100 mA cm^−2^ in (a) a blank 1.0 M KHCO_3_ solution and (b) a 1.0 M KHCO_3_ solution containing 0.05 M KI.

**Figure S11** e-CO_2_RR performance (FE for the generated products and operating potential) of the (a) GDE|Au and (b) GDE|Cu_2_O electrodes, obtained from 2-h electrolysis at -200 mA cm^−2^ in the 1.0 M KHCO_3_ solution containing 0.05 M KI.

**Figure S12** High-resolution I 3d XPS spectra of the (a) Au@Cu_2_O (*n*=2) and (b) Au@Cu_2_O (*n*=5) electrodes following 2-h electrolysis at -200 mA cm^−2^ in the 1.0 M KHCO_3_ solution containing 0.05 M KI.

**Figure S13** e-CO_2_RR performance of the GDE|Au@Cu_2_O (*n*=5) electrode, obtained from 24-h electrolysis at -200 mA cm^−2^ in the blank 1.0 M KHCO_3_ solution. (a) FE for the generated products and operating potential. (b) EE_C2+_.

**Figure S14** (a) TEM and (b) SEM images of the GDE|Au@Cu_2_O (*n*=5) electrode obtained following 24-h electrolysis at -200 mA cm^−2^ in the blank 1.0 M KHCO_3_ solution.

**Figure S15** Raman spectra of (i) the as-prepared GDE|Au@Cu_2_O (*n*=5) electrode and (ii) the GDE|Au@Cu_2_O (*n*=5) electrode obtained following 24-h electrolysis at -200 mA cm^−2^ in the blank 1.0 M KHCO_3_ solution.

**Figure S16** Post-electrolysis characterization of the GDE|Au@Cu_2_O (*n*=5) electrode following 24-h electrolysis at -200 mA cm^−2^ in the blank 1.0 M KHCO_3_ solution: (a) high-resolution Cu 2p XPS spectra, (b) Au 4f XPS spectra, (c) Cu LMM Auger spectra, and (d) relative content of surface copper species quantified from the XPS and AES analyses. Peak A in panel (c) denotes an auxiliary transition state within the Cu LMM spectrum.

**References**

1.	W. Xu, R. Xiao, S. An, C. Li, J. Ding, H. Chen, H. B. Yang and Y. Feng, “Engineering the Au-Cu_2_O Crystalline Interfaces for Structural and Catalytic Integration,” *Small* 19 (2023): e2300587, <https://doi.org/10.1002/smll.202300587>.

2.	C. Rettenmaier, A. Herzog, D. Casari, M. Ruscher, H. S. Jeon, D. Kordus, M. L. Luna, S. Kuhl, U. Hejral, E. M. Davis, S. W. Chee, J. Timoshenko, D. T. L. Alexander, A. Bergmann and B. R. Cuenya, “Operando insights into correlating CO coverage and Cu-Au alloying with the selectivity of Au NP-decorated Cu_2_O nanocubes during the electrocatalytic CO_2_ reduction,” *EES Catalysis* 2 (2024): 311-323, <https://doi.org/10.1039/d3ey00162h>.

3.	Z. Zhang, H. Guo, S. Li, S. Gao, X. Wang, M. Li, W. Yan and H. Xu, “Cavity-confined Au@Cu_2_O yolk-shell nanoreactors enable switchable CH_4_/C_2_H_4_ selectivity,” *Nature Communications* 16 (2025): 7559, <https://doi.org/10.1038/s41467-025-62875-8>.

4.	M. Wang, S. Zhang, M. Li, A. Han, X. Zhu, Q. Ge, J. Han and H. Wang, “Facile synthesis of hierarchical flower-like Ag/Cu_2_O and Au/Cu_2_O nanostructures and enhanced catalytic performance in electrochemical reduction of CO_2_,” *Frontiers of Chemical Science and Engineering* 14 (2020): 813-823, <https://doi.org/10.1007/s11705-019-1854-8>.

5.	W. Tan, B. Cao, W. Xiao, M. Zhang, S. Wang, S. Xie, D. Xie, F. Cheng, Q. Guo and P. Liu, “Electrochemical Reduction of CO_2_ on Hollow Cubic Cu_2_O@Au Nanocomposites,” *Nanoscale Research Letters* 14 (2019): 63, <https://doi.org/10.1186/s11671-019-2892-3>.

6.	S. Shen, X. Peng, L. Song, Y. Qiu, C. Li, L. Zhuo, J. He, J. Ren, X. Liu and J. Luo, “AuCu Alloy Nanoparticle Embedded Cu Submicrocone Arrays for Selective Conversion of CO_2_ to Ethanol,” *Small* 15 (2019): e1902229, <https://doi.org/10.1002/smll.201902229>.

7.	H. E. Shim, G. Yun, S. Y. Hwang, S. Y. Kim, C. K. Rhee and Y. Sohn, “Laser ablation-controlled Au/Cu interfaces for modulating C_1_, C_2_, and C_3+_ chemistry in electrochemical CO_2_ reduction,” *Materials Today Energy* 49 (2025): 101842, <https://doi.org/10.1016/j.mtener.2025.101842>.

8.	F. Wang, H. Tian, G. Chen, Z. Zhao, W. Tu, W. Jiang and G. Liu, “Engineering Au/Cu interfaces to promote *CO spillover for high C_2+_ selectivity in CO_2_ electroreduction,” *Journal of Materials Chemistry A* (2026), <https://doi.org/10.1039/d6ta01760f>.

9.	Y. Xu, X. Hu, J. Zhu, Z. Wang, X. Liu, J. Dai, J. Gong, H. Liu and G. Li, “Compositional gradient Au–Cu bimetallic heterostructures for efficient electroreduction of CO_2_ to ethanol at low potential,” *Journal of Materials Chemistry A* 13 (2025): 24706-24715, <https://doi.org/10.1039/d5ta03359d>.

10.	Y. Xu, J. Huang, D. Zhu, X. Hu, X. Liu, Z. Wang, G. Xu, J. Dai, J. Gong, H. Liu and G. Li, “AuCu bimetallic nanourchins with low-coordinated sites for highly efficient electroreduction of CO_2_ to acetate at low potential,” *Chemical Communications* 61 (2025): 14434-14437, <https://doi.org/10.1039/d5cc03660g>.
